# Assessing the effects of ROO and tariff margin on China-ASEAN free trade agreement utilization

**DOI:** 10.1371/journal.pone.0286106

**Published:** 2023-05-19

**Authors:** Yahui Qu, Ruxu Zhang

**Affiliations:** School of Economics, Beijing Technology and Business University, Beijing, China; University of Sargodha, PAKISTAN

## Abstract

This study investigates the effects of rule of origin (ROOs) and tariff margin on China-ASEAN Free Trade Agreement (CAFTA) utilization. Using a sample of 40,474 product-level observations with China’s imports from ASEAN countries during the period 2015 to 2021 and adopting the Logit model estimation methods, we found that larger tariff margin positively affects the use of CAFTA, whereas, the rules of origin show a negative effect on the CAFTA utilization. In order to assess the specific impact of two effects, we also calculate the relative contribution of these two effects to the CAFTA utilization by ASEAN countries, and the results show that the rules of origin play a more important role on the CAFTA utilization by each ASEAN country. Moreover, based on heterogeneity analysis, we also find that ROOs play an important role in the use of FTA by lower middle-income countries and the tariff margin play an important role in the use of FTA by upper middle-income and high-income countries. Based on the above findings, the study proposes some policy recommendations on how to increase the CAFTA utilization by reducing the ROO costs and accelerating tariff reductions.

## 1. Introduction

**Fig 1:**
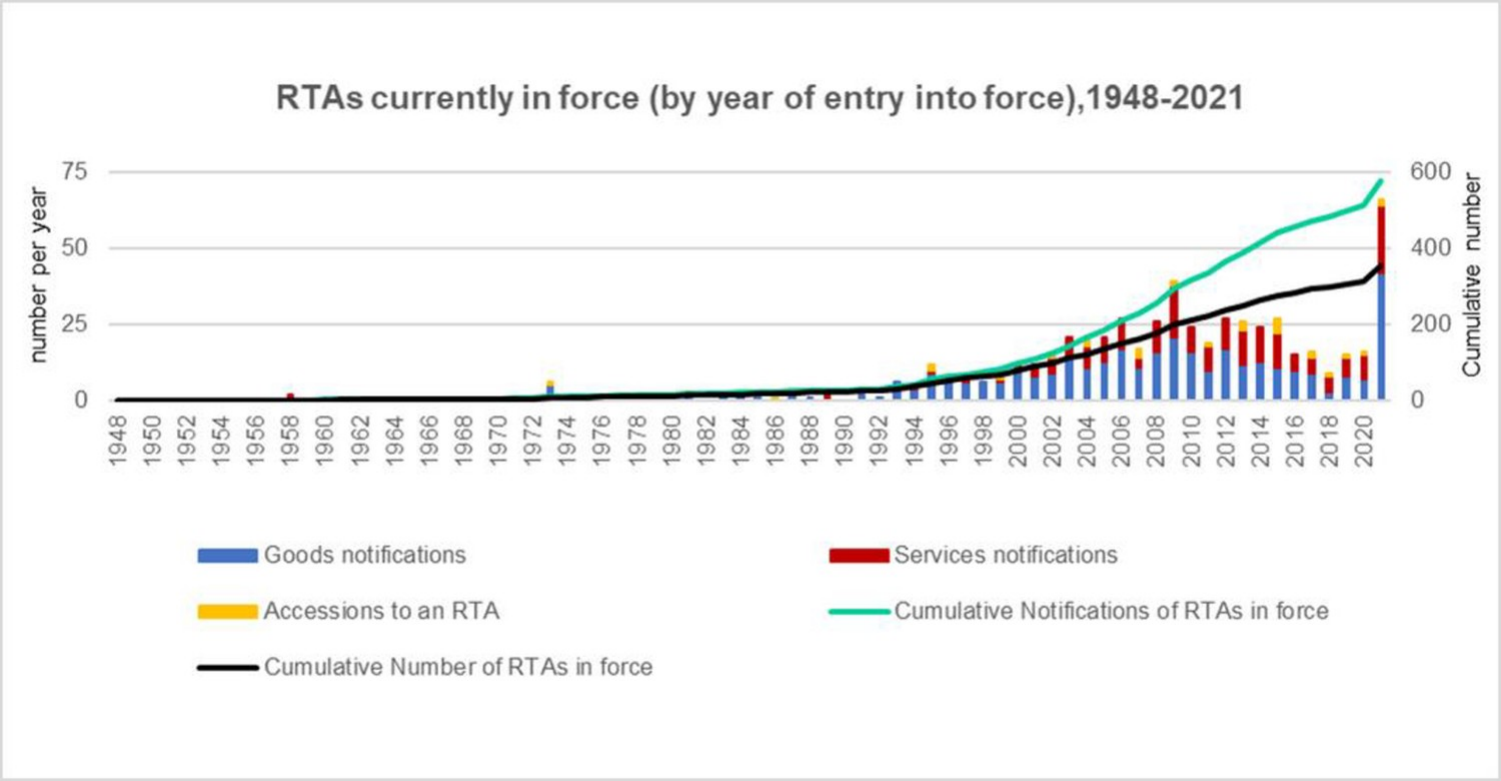
The growth of RTAs. RTAs is the abbreviation for Regional Trade Agreements. The goods, services & accessions to an RTA are counted separately. The cumulative lines show the number of RTAs/notifications currently in force from 1948 to 2021.

With the deepening of global value chain division, multinational cooperative production has become the mainstream trend of world trade development. In order to participate in the division of global value chain and enter the world market, many countries choose regional trade agreements (RTAs) as the main means of economic cooperation. Thus, the number of RTAs has increased rapidly since the mid-1990s. As of December 2021, the World Trade Organization (WTO) reported that 354 RTAs were in force and a further 579 RTAs were under negotiation (see Fig 1). Along with the proliferation of regional trade agreements worldwide, as the most common form of RTAs, free trade agreements (FTAs) are gradually becoming an important negotiating item in the development of foreign trade for many countries since it can enhance their access to the world market. However, despite this proliferation, many exporters make a low use of FTAs, either underutilize FTAs or ignore entirely not to use them, where, the rules of origin are often identified as the primary reason why FTAs are underutilized [1]. As with any other preferential program, FTA requires that products comply with certain rules of origin, which specify the conditions that must be satisfied for a product to be considered of origin [2]. As a result, the importance of ROOs has been increasing over the last few years as more countries engage in FTAs and begin to grant specific trade preferences or restrictions to imported goods based on where the product is produced and when the origin is determined [3].

As a key component of any free trade agreements, ROOs are originally developed to prevent trade deflection by non-member countries due to free-riding. However, with the establishment and implementation of FTAs, the overly stringent ROOs have become an instrument for trade protection, seriously affecting the development of trade between member countries and the use of FTA. In order to enjoy preferential treatment under FTA, exporters must satisfy the ROO requirements. For this, they must make a ‘substantially’ transform on the product, or provide a ‘significant’ contribution to regional value content, or even both. In particular, exporters may increase their shares of either local inputs or imports from FTA partners in total inputs under the requirement of regional value content criteria [4]. Thus, exporters have to pay the costs imposed by ROOs. The stricter ROOs, this cost-raising effect for exporters is higher [5], and then the lower the use of FTA by exporters. And tariff preferences, as the benefits that exporters derive from the FTA utilization, also have a greater impact on the use of FTA. Generally speaking, the larger the tariff margin, the greater the benefits, and then the higher the use of FTA by exporters.

As the first free trade area established by China to the outside world, the China-ASEAN Free Trade Area has achieved breakthrough growth in trade between the two sides since it was fully established in 2010. In particular, the scale of bilateral trade between China and ASEAN grew at an average annual rate of 9.4 percent from 2010 to 2019. The signing and entry into force of the Upgrading Protocol has further unlocked the economic and trade potential within the FTA. The upgraded agreement has enriched, improved, supplemented and enhanced the content covering the areas of trade in goods, trade in services, investment, and economic and technical cooperation. In particular, in the area of trade in goods, both sides have optimized rules of origin and trade facilitation measures, and the coverage of zero-tariff products has reached over 90 percent. As an example of regional economic cooperation in East Asia, the upgrading and use of China-ASEAN FTA not only concerns the further deepening of bilateral economic and trade relations, but also plays a vital role in the rapid development of regional economic cooperation in Asia and global. Therefore, it is necessary to explore and analyze in depth the use of CAFTA.

To this end, based on the two important factors affecting FTA utilization mentioned above, this paper assesses the effects of ROO and tariff margins on the CAFTA utilization. The main contributions of this study are as follows: First, we calculate the relative contribution of ROO effect and tariff margin effect to the CAFTA utilization by ASEAN countries based on the average marginal effect of variables. It was found that ROOs play a greater role in the CAFTA utilization than tariff margin. Second, this paper examines the effects of ROO and tariff margin on the CAFTA utilization across countries with different income levels. The results show that ROO has a more significant impact on the CAFTA utilization in lower middle-income countries, while the tariff margin has a more significant impact on the CAFTA utilization in upper middle-income and high-income countries. Third, based on the findings of this paper, we propose policy recommendations related to increasing the use of CAFTA by reducing the ROO costs and accelerating tariff reductions.

The rest of this paper is organized as follows. Section 2 is the literature review and theoretical framework of this study. Section 3 introduces the materials and methods. Section 4 shows the regression results and discussion of this paper. Section 5 concludes this study.

## 2. Literature review and theoretical framework

### 2.1. Literature review

With recent proliferation of free trade agreements, numerous scholars have conducted the theoretical and empirical research on FTAs. We can divide the literature most relevant to this paper into three groups and summarize them as follows.

First, scholars have studied the determinants of FTA formation (Sapir, Baldwin, Mansfield et al.) and its trade effects (Tinbergen, Aitken et al., Brabalet et al., Frankel et al., Ghosh et al.), Baier and Bergstrand argue that the likelihood of an FTA is higher, the more different the labor input factor ratios based on Heckscher-Ohlin trade [6]. Viner first provides the trade creation effect and trade division effect on FTA, and point that a regional trading agreement is beneficial (harmful) depended on the magnitude of trade creation is larger (smaller) than trade diversion [7]. Baier and Bergstrand use the standard cross-section gravity equation to estimate that the average free trade agreement approximately doubles bilateral trade after ten years [8]. Dai et al. argue that FTAs do divert FTA member imports away from non-member countries by extending the econometric specification of the structural gravity model to estimate the trade diversion effect of FTA [9]. Barbalet et al. show that PTAs generally increase trade between members but there are often offsetting negative effects on trade with non-members by analyzing effects of 27 agreements that are of particular importance for Australia on the value of merchandise trade flows [10]. Russ and Swenson estimate the value of trade diversion in the two years following the implementation of the Korea-U.S. FTA at $13.1 billion in 2013 and $13.8 billion in 2014, and that the U.S. bilateral goods trade deficit with South Korea increased at roughly the same rate as the magnitude of trade diversion [11].

Second, the utilization rate of free trade agreement as an important indicator measuring the efficiency of free trade area, many studies show the key elements on affecting the use of FTA. ROOs as the first important factor, Krishna analyzes the cost of ROOs in FTAs based on the factor input model of Heckscher-Ohlin theory. Céline et al. argue that the lower utilization rate of NAFTA mainly attribute to the presumed cost-raising effects of these seemingly ‘made-to-measure’ ROO [12]. Malkawi et al. have examined the extent to which ROOs still represents an obstacle to the full implementation of GAFTA [13]. Mukunoki and Hirofumi argue that if the threshold required by ROO is too high, even if all trade within the FTA is duty-free, the impact of the aggregate effect may still be negative [14]. Sytsma found that the relaxing rules of origin can allow for more foreign content in exported products has a substantial effect on preference utilization rates [15]. Another important factor is tariff reduction, Medalla (2009) argues that the lower tariff preference is also one important reason for the low utilization of AFTA, as most ASEAN countries have preferences below the 5% threshold [3]. Kim and Cho show that higher tariff margin promotes the use of Korea-ASEAN FTA, but the impact of the average value of imports per-application is not significant by using restrictive index, margin of preferences and other relevant variables [16]. Furthermore, other studies examined the effect of both factors simultaneously. Athukorala and Kohpaiboon [17], Hayakawa argue that small preference margins and the ROO costs are the most common reasons for the non-use of FTAs [18]. Hayakawa et al. explore the determinants on utilization of the Korea-ASEAN free trade agreement, and showed that restrictive ROOs have a negative effect on FTA utilization while the tariff margin and average trade have a positive effect on it [19]. Based on the findings of Nag and De [20], in the context of global value chain division of labor, the amplifying effect of production costs makes firms more sensitive to the compliance cost of rules of origin. Therefore, Guo point that a small increase in the degree of restriction of rules of origin may lead to a significant increase in the compliance cost of rules of origin, thus reducing firms’ utilization of preferential tariffs [21].

Third, some studies have even computed the specific cost of FTA utilization (Francois et al., Cadot and de Melo, Ulloa and Wagner, Cherkashin et al., Hayakawa et al.). For example, Francois et al. calculated the erosion of trade preferences that equal to an average of 4 percent of the value of goods trade [22]. Hayakawa showed that the average tariff equivalent of fixed costs of using of FTA for all existing FTAs in the world is estimated to be around 3 percent by employing the threshold regression method [23]. Cherkashin et al. structurally estimated the documentation costs of ROO compliance, which were USD 4,240 [24]. Hayakawa et al. even have measured the cost of FTA utilization for exporting from China and Korea to Thailand, and found that the median costs for FTA utilization are estimated to be around two thousand US dollars in the case of exporting from China and around one thousand US dollars in the case of exporting from Korea [25].

As mentioned above, scholars have done extensive research on the FTA utilization and achieved certain results. However, the impact on the China-ASEAN FTA utilization has never been addressed in these studies, especially the rules of origin and tariff margin, which are considered to be two important factors affecting the use of FTA. For this, this paper assesses the effects of ROO and tariff margin on the CAFTA utilization using product-level data on China’s imports from ASEAN countries.

### 2.2. Theoretical framework

This study is based on H-O theory and draws on Krishna’s [26] cost analysis model on ROO. According to the Heckscher-Ohlin (H-O) theory, each country exports goods that are produced intensively using its relatively abundant factor endowment. Within an FTA, member countries can use their respective factor endowments to produce products in which they have a comparative advantage, and then export them to other member countries using the tariff preferences granted by the FTA. That is, member countries in the FTA maximize their own benefits at the lowest possible cost, thereby increasing their own welfare levels.

**Fig 2:**
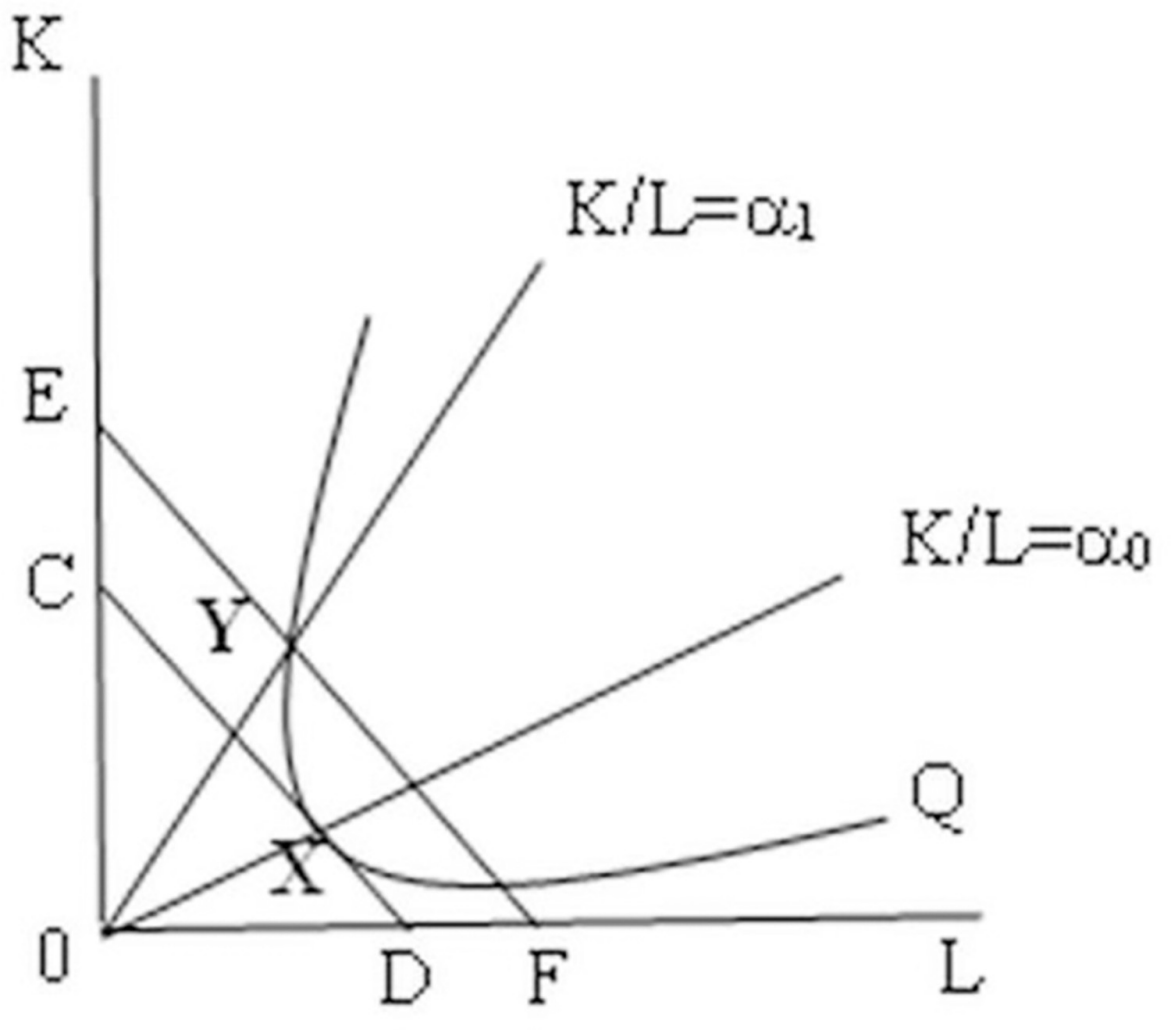
The ROOs and costs. Q represents the iso-quant line curve, CD and EF represent the iso-cost line, and points X and Y represent the cost of producing the same amount of product. point X is the cost without the rules of origin (ROOs), and point Y is the cost after the introduction of ROOs. It is clear that the cost represented by point Y is greater than the cost represented by point X. This indicates that meeting the requirements of ROOs increases the cost of production.

Fig 2 illustrates the costs incurred to meet the rules of origin requirements. We assume that member country A of the FTA make the product with inputs (L) and (K) under constant returns to scale. In the absence of rules of origin and maintaining factor prices constant, the country A chooses to produce the factor input combination represented by the point where the iso-cost line CD is tangent to the iso-quant line curve Q, i.e., the lowest production cost at point X. At this point, the ratio of K to L is equal to α_0_. If the rule of origin is used and it requires the ratio of K to L to be equal to α_1_, α_1_ must be greater than α_0_. Then only the point along the iso-quant line curve Q and to the upper left of point X meets the requirement. Therefore, production is carried out according to the combination of inputs minimized at point Y. The cost line is represented by the height of line EF. It is clear that the cost represented by line EF is greater than that of line CD, indicating that the ROOs increase the cost of country A, making the benefit from production at point Y smaller than the benefit from production at point X. If the cost of rules of origin is represented by R (α_1_), then the ratio a of K to L will keep getting larger as the degree of restriction of ROO increases, and thus R (α_1_) will also keep getting larger. The greater the cost of meeting the rules of origin, the smaller the benefit obtained by country A.

**Fig 3:**
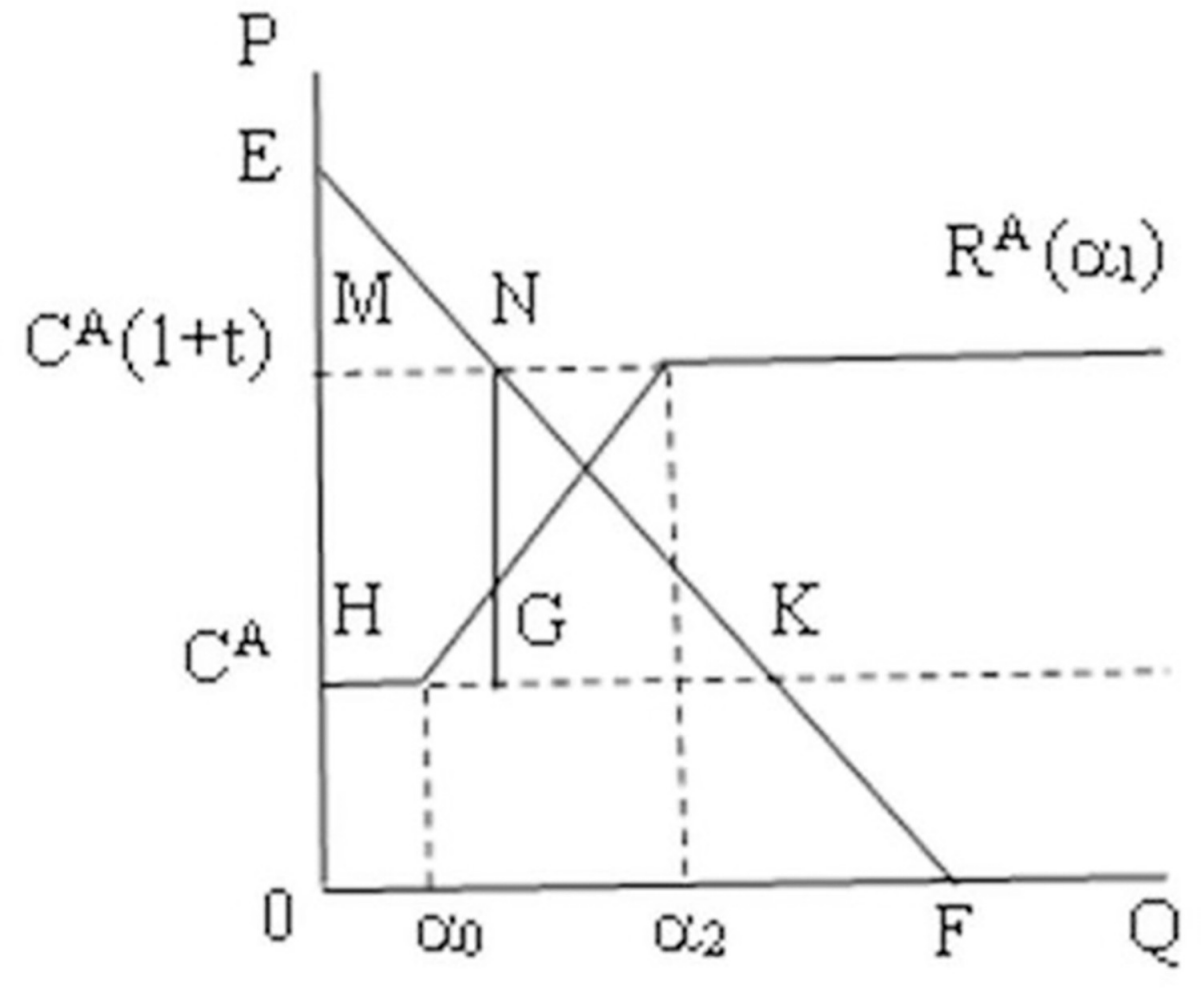
The ROOs costs of using FTA. C^A^ denotes the cost before the signing of the FTA and C^A^(1+t) denotes the total cost of the exported product before signing the FTA, including the production cost and the tariff cost. The rules of origin are introduced after signing the FTA, R^A^ (α_1_) denotes the cost of satisfying ROOs, Country A will continue to use the FTA as long as R^A^ (α_1_) is less than C^A^(1+t), i.e. MNHK is greater than MNHG. Conversely, will abandon the use of FTA.

Based on the analysis of ROOs cost in Figs 2 and 3 compares and analyzes the cost of using FTA by member country A. The production cost is C^A^ before signing the FTA, when α_1_ is less than or equal to α_0_, R^A^ (α_1_) = C^A^. R^A^ (α_1_) increases with α_1_ after signing, but as long as the consumer surplus (MNHK) is greater than the tariff revenue (MNHG), and α_0_<α_1<_α_2_, the ROO costs are acceptable. In other words, member country A will continue to use FTA as long as the benefits from the tariff preferences granted by the FTA are greater than the costs paid to meet the rules of origin. When α_1_ is more than or equal to α_2_, R^A^ (α_1_) equal to the tariff cost before signing FTA, i.e., R^A^ (α_1_) = C^A^(1+t), at this time, the benefits from the tariff preferences granted by the FTA are less than the costs of meeting the rules of origin, then member country A will give up using FTA. Through the above theoretical analysis, tariff preference as the benefit gained by member country A using FTA, and rules of origin as the cost paid by member country A using FTA, only when the benefit is larger and the cost is smaller, member country A will choose to use FTA, otherwise it will give up using FTA. therefore, this paper proposes the following hypothesis:

H1: The rules of origin have a negative effect on the FTA utilization.

H2: The tariff preferences have a positive effect on the FTA utilization.

## 3. Materials and methods

### 3.1. The trade development China-ASEAN trade

**Fig 4:**
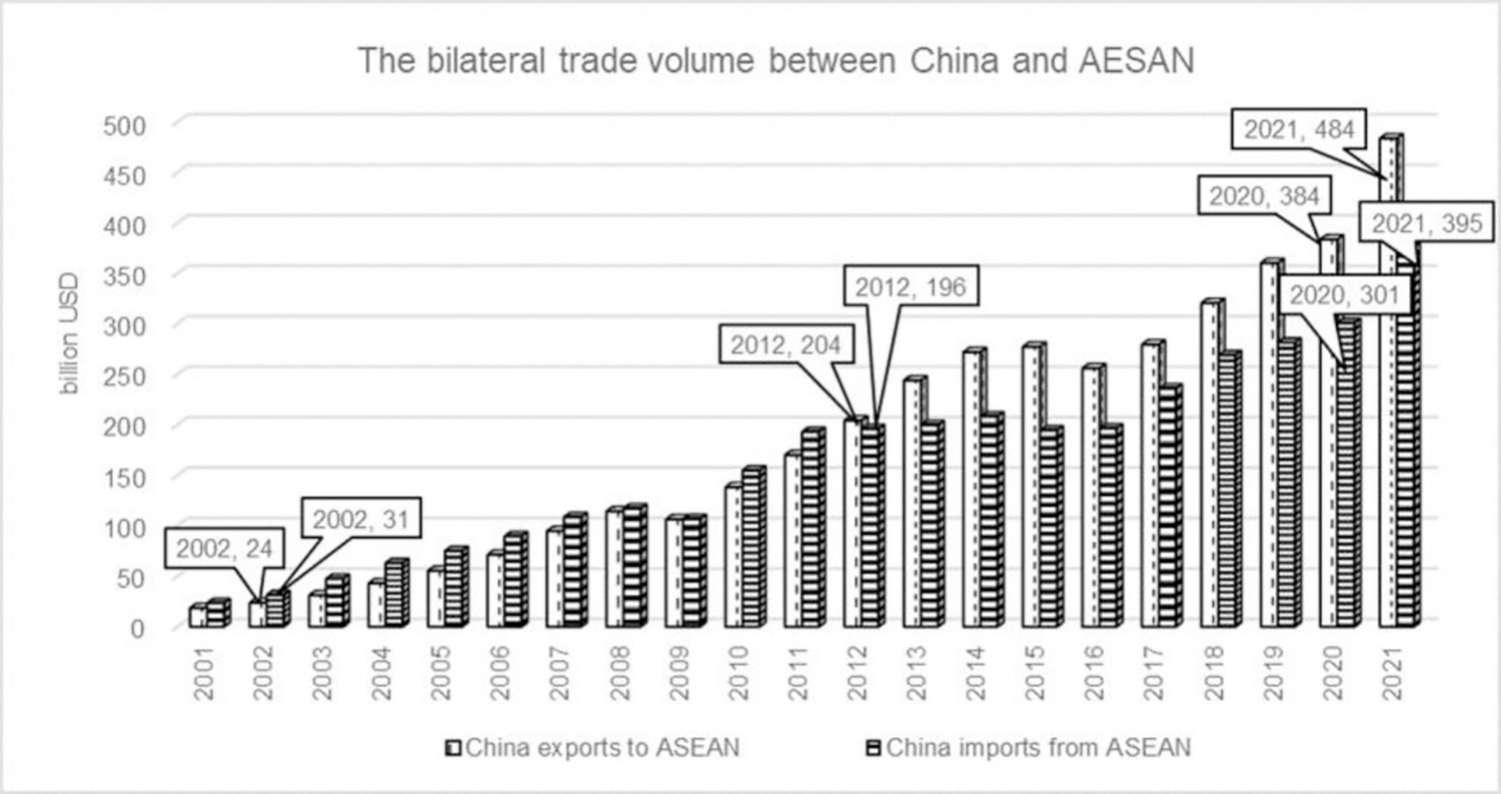
The trade volume between China and ASEAN. The white bar chart on the left represents China’s exports to ASEAN from 2002 to 2021, while the black bar chart on the right represents China’s imports from ASEAN from 2002 to 2021. China and ASEAN signed Free Trade Agreement in 2002. In 2012, China’s exports to ASEAN exceeded China’s imports from ASEAN for the first time, with bilateral trade still bucking the trend in 2020 and 2021, unaffected by the epidemic.

As of December, 2021, China has signed nineteen FTAs in place with twenty-six countries and regions. Among these FTAs, the China-ASEAN FTA is the first and largest free trade area established by China, which has made fruitful achievements since it was fully completed in 2010. The bilateral trade scale between China and ASEAN enlarged continuously since 2001. In 2002, when the CAFTA was just started, the bilateral trade volume was USD 54.8 billion. By 2012, the bilateral trade volume rose up to USD 400.1 billion, increasing eight times during the ten years with an annual growth of 19.8 percent, and even China’s exports to ASEAN exceeded its imports from ASEAN at the first time. Moreover, bilateral trade and economic cooperation has been accelerated on a fast track since the upgraded FTA came into force on July 1, 2016. In particular, the trade volume still grew bucking the trend even in the midst of the 2020 global epidemic and reached USD 684.6 billion, which making a historic breakthrough of becoming each other’s largest trading partner. The growth trend in is still maintained 2021, in which China’s import and export trade with ASEAN increased by 31.1 percent and 26.1 percent respectively (see Fig 4).

### 3.2. The CAFTA utilization by ASEAN countries

The liberalization level of trade in goods of the existing China-ASEAN FTA is very high. Almost 90 percent of the products of both sides have realized zero tariffs [27]. Therefore, in the upgrading protocol, both sides pay more attention to the facilitation of trade in goods. Specifically, both sides optimized and improved the rule of origin, further simplified the customs clearance procedure, promised to provide enterprises of both sides with high-efficient and rapid clearance service by taking measures such as automatic system and risk management and increased the use of CAFTA by exporters.

**Fig 5:**
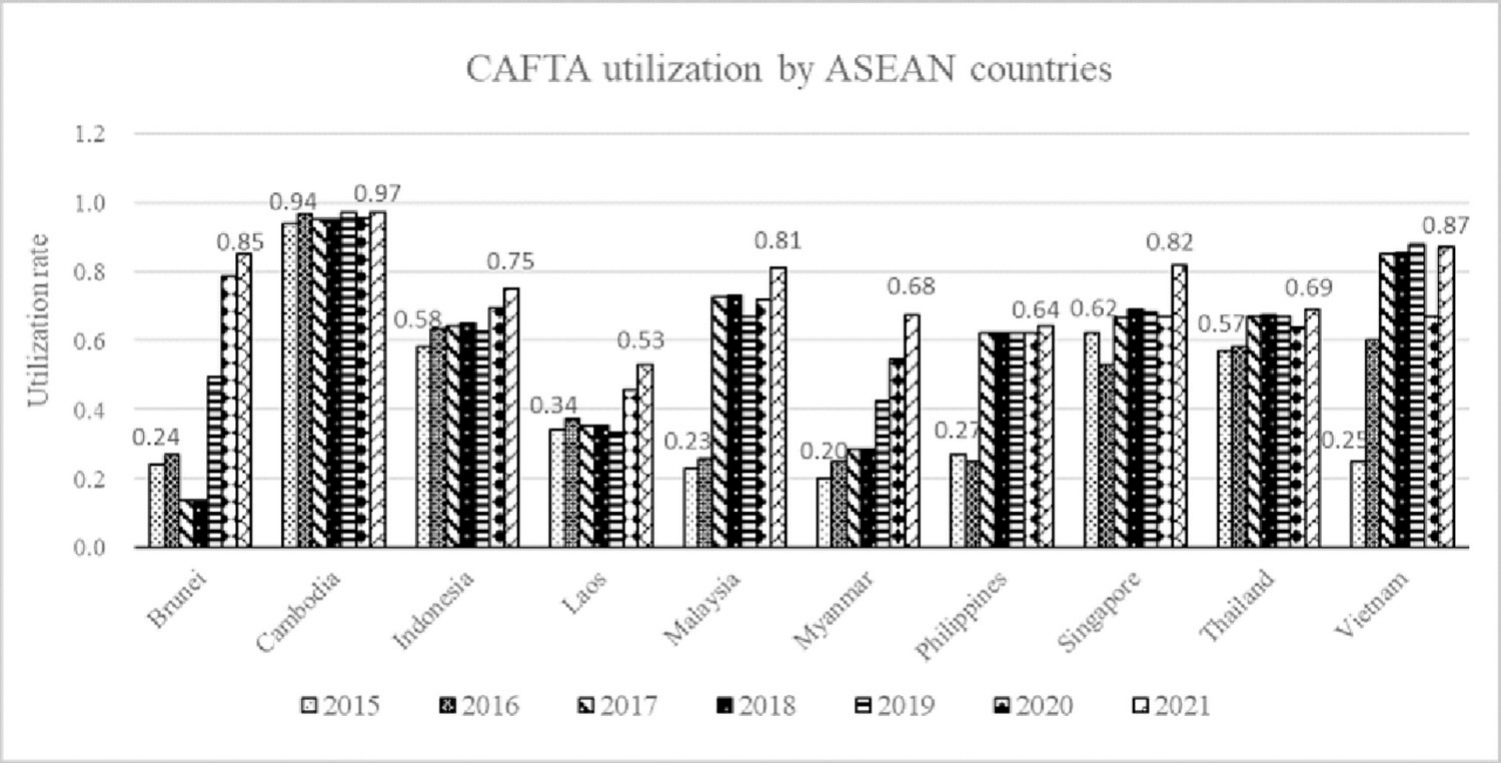
The CAFTA utilization rate by exporting country. The seven-pattern bar chart represent the utilization of CAFTA by ASEAN countries when exporting to China from 2015 to 2021, and also mark the utilization rate of CAFTA for the two years 2015 and 2021.

As the upgraded China-ASEAN FTA constantly improves the detailed rules and requirements of various fields in the agreement, ASEAN countries increase the use of CAFTA when exporting to China. Fig 5 shows the utilization rate of CAFTA in ASEAN member countries from 2015 to 2021. On the whole, the use of CAFTA in ASEAN countries has been increasing during the seven years, except for individual countries that may be shocked by the epidemic in 2020. Specially, Vietnam is considered as the largest increase on the CAFTA utilization, increasing more than two times from 0.25 in 2015 to 0.87 in 2021.

### 3.3. Econometric model

The purpose of this section is model specification for empirical analysis, specifically, to identify the effects of ROO and tariff margin on the use of CAFTA. Based on the gravity model, this study constructs an empirical framework where the dependent variable is utilization rate to explore the determinants on the CAFTA utilization. However, given that preferential tariffs on most products have been reduced to zero since 2015 and that MFN tariffs generally do not change too much, thus leading to the estimated results for the tariff preference margin may not be significant. Therefore, we choose to use MFN instead of tariff preference margin and employ the following two regression equations:

$${UR}_{ixt}=\beta_{0}+\beta_{1}{RI}_{ixt}+\beta_{2}{Margin}_{ixt}+\beta_{3}{CGDP}_{ixt}+\beta_{4}{AGDP}_{ixt}+\beta_{5}{Distance}_{ixt}+\beta_{6}\gamma_{ind}*{year}_{t}+\varepsilon_{ixt}$$ (1)

$${UR}_{ixt}=\beta_{0}+\beta_{1}{RI}_{ixt}+\beta_{2}{MFN}_{ixt}+\beta_{3}{CGDP}_{ixt}+\beta_{4}{AGDP}_{ixt}+\beta_{5}{Distance}_{ixt}+{\beta_{6}\gamma }_{ind}*{year}_{t}+\varepsilon_{ixt}$$ (2)

where *UR*_*xit*_ denotes the CAFTA utilization rate by product x of each ASEAN exporting country i in year t, *Margin*_*ixt*_ indicates the tariff preference margin in year t for product x of each ASEAN exporting country i under the China-ASEAN FTA, *MFN*_*ixt*_ is China’s most favor nation tariff rate for product x of ASEAN exporting country i in year t, *RI*_*ixt*_ is a dummy variable, indicating the restrictiveness index of rules of origin. In addition, the equations also introduce the control variables. *CGDP*_*ixt*_ is the GDP value of the importer i in year t, *AGDP*_*ixt*_ is the GDP value of each ASEAN exporter i in year t. *Distance*_*ixt*_ is the distance between the two capitals of ASEAN exporting country i and China, *γ*_ind_*year_*t*_ is the interaction term of industry fixed effect and time fixed effect, ε_*ixt*_ is the error term.

By the above calculation, the utilization rate of CAFTA takes the value of zero or one. In view of the data characteristic of the dependent variable, we choose the logit estimation method that can be used for the binary choice model. Based on the hypothesis presented in this paper, as in previous studies, we expect that the ROOs have a negative effect on the CAFTA utilization, while the tariff margin have a positive effect on the CAFTA utilization.

### 3.4. Sample data and variables source

**Fig 6:**
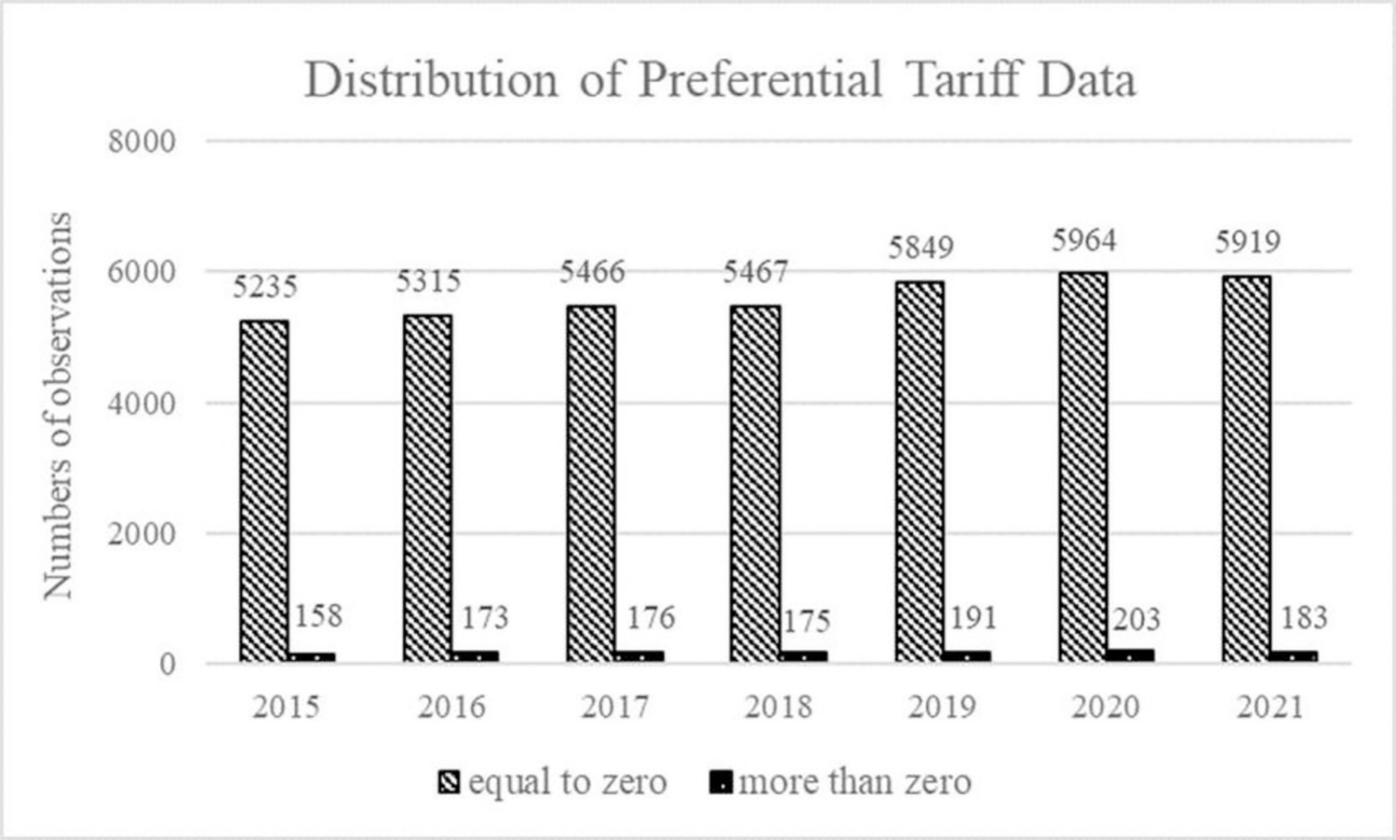
The distribution of preferential tariff data. The data on preferential tariffs for products from 2015 to 2021 are all greater than or equal to zero. The bar chart on the left denotes the number of products with preferential tariffs equal to zero, and the bar chart on the right denotes the number of products with preferential tariffs greater than zero. It is clear that the number of products with a preferential tariff equal to zero is very high, both exceeding 90%.

The study takes products at the HS 6-digit level from the product specific rules of origin specified in goods trade as the research subject. For this, we focus on the utilization of CAFTA in goods trade imports, especially China imports from ASEAN member countries, the initial sample consists of 63,294 observations. Where, these observations that the trade value is null and the tariff margin is null or less than zero for correspond year are excluded from sample. So, the final sample consists of unbalanced panel data, and the effective sample for our study is 40,474 observations at the HS 6-digit level from 2015 to 2021.It should be noted that the number of these observations with preferential tariff rates equal to zero is 39,215 and the number of non-zero is 1,259. This may have an impact on the effect of tariff margin, since the tariff margin is equal to the difference between MFN rates and preferential rates. Fig 6 shows the distribution of preferential tariff data, indicating that the preferential tariff on most products have been reduced to zero since 2015.

In order to assess the ROO effect and margin effect on the CAFTA utilization, the study uses ROOs restrictive index and tariff margin as kernel variables. For the rule of origin, it is almost impossible to find an instrumental variable that is strictly exogenous and can replace it, so we include the interaction term of fixed-effect in the model to reduce the impact of the rule of origin variable due to the endogeneity problem. In addition, to prevent endogeneity problems due to omitted variables, the paper also introduces the control variables, which are respectively CGDP, AGDP and Distance. Then empirically investigating the relation between CAFTA utilization and related variables.

#### 3.4.1. Independent variables

*3*.*4*.*1*.*1*. *Rules of origin-ROOs*. The ROOs restrictiveness index is constructed at the HS 6-digit level, since ROOs in the CAFTA were negotiated at the 6-digit level of the Harmonized System (HS). The data of rules of origin are obtained from the text of China-ASEAN Free Trade Agreement on the China Free Trade Area Service Network. A restrictiveness analysis obtains significant meaning in terms of analyzing present conditions on utilizing rules of origin for FTA. So, as for the measurement of the restrictive index of rules of origin, this paper mainly adopts the seven-point system proposed by Estevadeordal [28]. Estevadeordal’s seven-point assignment method (which, as noted above, take values in the range between one and seven), the higher the restrictiveness index is, the more stringent the rules of origin are. Among them, the rules of Wholly Obtained and Change in Tariff Chapter are given a higher score, while the rules of Regional Value Content and Change in Tariff Sub-heading are given a lower score.

We have compiled and summarized the product-specific rules of origin in the China-ASEAN Free Trade Agreement. Considering the strictness and complexity of ROOs under CAFTA, which includes single criteria, selective criteria and composite criteria, the paper makes some appropriate adjustment to the assignment of ROOs based on the improvement of the seven-point system proposed by previous studies [29]. The composite criteria are more stringent than the single criteria, and the single criteria is more stringent than the selective criteria. Obviously, the selective criteria have a greater flexibility when comparing with these three criteria. Therefore, the value of single criteria is normally assigned according to the previous research. For the composite criteria, the value equals to the sum of the two rules, while the value of selective criteria equal to the average score of the sum of the two rules minus one. Where, when the single criteria and composite criteria are combined to form the selective criteria, the value equals to the average of the two rules subtract a quarter. Based on the point system described above, the resulting restrictiveness index (RI) is assigned a single value, ranking from 1 to 7 (1 ≤RI≤ 7), representing the severity of ROO.

There are fourteen types of ROOs used for the CAFTA and the score of these types are reported in Table 1. Furthermore, the third column of Table 1 also summarizes the frequency use of different ROO types for CAFTA. As we can see, the selective criteria are used more frequently, accounting for 72 percent in the total number of (6-digit) HS tariff lines, most commonly the selective rule of CTH (Change in Tariff Heading) or RVC (40), which cover 574 tariff lines. This is followed by the single rule of CTC (Change in Tariff Chapter), applying for 352 tariff lines. Based on Table 1, we calculate the ROO restrictiveness index of the China-ASEAN FTA is equal to 3.5, so the ROO dummy variable takes on a value of one when RI is greater than or equal to 4 and zero otherwise.

**Table 1:** ROO types in the China-ASEAN FTA (6-digit HS lines).

Criteria Type	ROO Types	score	Frequency
Single Criteria	WO	7	247
	CTC	5	352
	CTH	4	3
	RVC 40	4	52
Selective Criteria	CTC or RVC 40	3.5	218
	RVC 40 or CTH	3	574
	RVC 40 or CTSH	2	133
	RVC 40 or Process Rule 1	3	49
	RVC 40 or Process Rule 2	3.25	78
	RVC 40 or CTH or Process Rule 3	3.5	269
	RVC 40 or CTH+WO	6.5	1
	RVC 40 or CTH+EXH	4.25	43
	RVC 40 or CTSH+EXSH	3	1
Composite Criteria	CTC+EXC	7	25
Total			2405

Abbreviations are as follows: Process Rule 1,2,3(a different procedure in the production process), CTC-Change in Tariff Chapter (2-digit), CTH-Change in Tariff Heading (4-digit), CTSH-Change in Tariff Sub-heading (6-digit), RVC (Regional Value Content), WO (Wholly Obtained), and EX (exception). Source: Authors’ calculations based on Product Specific Rules of Origin from the CAFTA text.

*3*.*4*.*1*.*2*. *Tariff margin-margin*. The tariff margin is constructed at the HS 6-digit level by using MFN tariff rates and preferential tariff rates, which are obtained from the World Integrated Trade Solution (WITS) [30] for the corresponding year. The tariff preference margin equals to the difference between the MFN tariff rates and the preferential tariff rates, where, the values that equal to null or less than zero are excluded from the data. In this regard, this paper expects a positive relationship between the tariff margin and FTA utilization.

*3*.*4*.*1*.*3*. *GDP-CGDP and AGDP*. The annual GDP in China and ASEAN countries is calculated by using current dollars, and reflects the economic size of both countries. The data is obtained from the World Bank.

*3*.*4*.*1*.*4*. *Distance between China and ASEAN capitals-distance*. Distance is a control variable and reflects the cost of transportation for trade between the two countries. We still get data from the World Bank.

#### 3.4.2. Dependent variables

In the study, we take the CAFTA utilization by ASEAN countries based on product level as dependent variables. Considering our dataset is for import data at the HS 6-digit codes, referring to the study by Keck and Lendle [31], using the definition of preference utilization, for product x (at tariff-line level) from exporting country i, we have:

$$u_{i,x}=\frac{{pref}_{x,i}}{{elig}_{x,i}}$$

**Fig 7:**
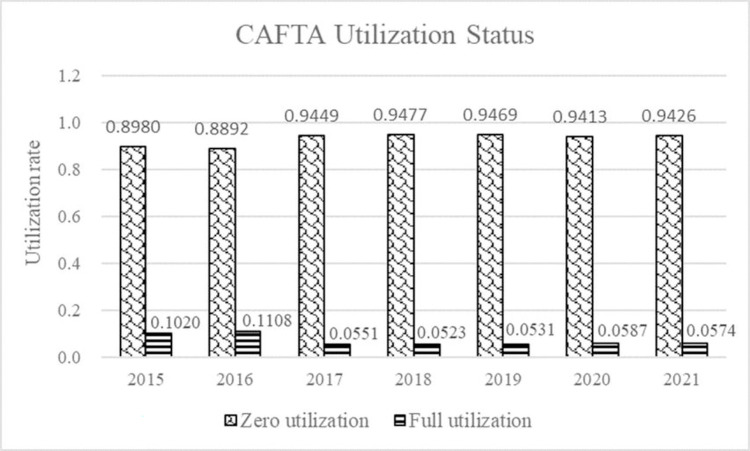
The CAFTA utilization status by ASEAN countries. The utilization status of CAFTA when ASEAN exporting to China from 2015 to 2021. The bar chart on the left indicates the rate of zero utilization and the bar chart on the right indicates the rate of full utilization. It is clear that the rate of zero utilization is greater than the rate of full utilization, indicating a low level of utilization of CAFTA by ASEAN countries.

For an individual transaction, the u is either 0 or 1. Similarly, the utilization rate of CAFTA by each ASEAN country is also calculated at the HS 6-digit codes. Base on the WITs database, we obtained the trade data at the HS 6-digit level under preferential tariff and MFN tariff treatment, as a proxy of the trade value enjoyed for preferential treatment and that eligible for preferential treatment, respectively. Thus, the value of CAFTA utilization is also 0 or 1 in our study. This value shows how effectively the FTA is utilized. Where, zero indicates FTA is not being utilized at all, one indicates FTA has achieved perfect utilization. Fig 7 shows the utilization status of CAFTA in China imports from ASEAN countries.

#### 3.4.3. Descriptive statistics

Table 2 reports the descriptive statistics for the variables used in the study, which displays mean, minimum, maximum and standard deviation (SD). They all were derived based on adding one to the variable and then taking the natural logarithm except for the dummy variable RI. It can be observed that the mean value of UR is 0.931, the minimum value is 0 and maximum value is 1, this means that ASEAN countries have a high utilization rate of CAFTA when exporting to China. The dummy variable with a minimum value of 0 and a maximum value of 1. The maximum value of tariff margin is 3.77 and the mean value is larger, equal to 1.28, meaning that the tariff margin may have a greater effect on the use of CAFTA. That is, the use of CAFTA will increase with the tariff margin.

**Table 2:** Summary statistics of the variables.

Variables	Observations	Mean	SD	Minimum	Maximum
UR	40,474	0.931	0.254	0	1
RI	40,474	0.147	0.354	0	1
MFN	40,474	1.35	1.188	0	3.77
Margin	40,474	1.28	1.18	0	3.77
CGDP	40,474	23.32	0.155	23.13	23.6
AGDP	40,474	19.56	0.98	16.25	20.89
Distance	40,474	8.18	0.269	7.75	8.56

The table reports number of observations (N), mean (M), standard deviation (SD), minimum and maximum of all the observations used in the study.

#### 3.4.4. Correlation analysis

Before estimating the regression equation, the study checks the correlation among the kernel variables. Table 3, presents the coefficient estimates of the correlation matrix. The outcome of the matrix indicates that UR is significant and positively correlated with tariff margin but negatively correlated with RI. Also, there is no sever correlation among the explanatory variables. This study further used variance inflation factor to examine whether multicollinearity exist or not in the model and the average variance inflation factor score is 1.14, which is lower than 5 which indicate that model is free from the multicollinearity problems.

**Table 3:** Correlation matrix.

Variables	UR	RI	MFN	Margin	CGDP	AGDP	Distance
UR	1.0000						
RI	-0.0044	1.0000					
MFN	0.2013***	0.2023***	1.0000				
Margin	0.2959***	0.1150*	0.9381***	1.0000			
CGDP	0.0644***	0.0112**	-0.2688***	-0.2675*	1.0000		
AGDP	-0.0497***	0.0238***	0.2615***	0.2737***	0.0501***	1.0000	
Distance	-0.0553***	0.0097*	0.1808***	0.1868***	-0.0217***	0.3633***	1.0000

***Significant at 1%

**Significant at 5%

*Significant at 10%.

#### 3.4.5. Unit root test

Prior to the empirical analysis, we performed a unit root test to verify the stationarity of the serials. Therefore, we choose to use the Im-Pesaran-Shin test based on panel characteristics and applicability. Because the normality of the Z-tild-bar in the IPS test requires that each group of observations be continuous variables with balanced data and a time trend, Table 4 only shows the results of the unit root test of the Margin and MFN variables. The p-value is equal to zero, indicating that the serials are stable.

**Table 4:** Im-Pesaran-Shin test.

Variables	Statistic	P-value
Margin	-9.1988	0.0000
MFN	-9.3135	0.0000

## 4. Results and discussion

### 4.1. Baseline regression analysis

Table 5 reports the estimated result of regression equation using the utilization rate as dependent variable, which employ the Logit model estimation methods. The column (1) shows that the estimated coefficient on RI is negative and significant at the 1% level. However, the variable tariff margin is omitted. As mentioned above, we guessed that it may be because the preferential tariff of most products has been reduced to zero since 2015 and the MFN tariff generally does not change too much, which results in a small or zero change in the tariff margin. Furthermore, if the preferential tariff equals to zero, the change in the tariff margin depends mainly on the MFN tariff, and the higher the MFN tariff, the larger the tariff margin. For this, we estimate Eq (2) in the same way using the MFN tariff instead of the tariff margin. The column (2) shows the estimation results of Eq (2). As with column (1), the dummy variable RI is negatively correlated with the utilization rate and also significant at the 1% level, indicating that rules of origin have a binding effect on the use of CAFTA. The difference is that MFN tariff has a positive effect and significant at the 1% level, this means that tariff margin does have a positive relationship with CAFTA utilization rate and contribute to some extent to the use of CAFTA by ASEAN countries. As a result, the coefficients of two core explanatory variables are exactly consistent with expected sign. Moreover, the control variables are also significant at 1% level but with different signs. Of these, CGDP promotes the use of CAFTA when ASEAN exports, meaning that the larger the economy, the higher the likelihood of the CAFTA utilization. The AGDP is just on the opposite, which has a negative relationship with CAFTA utilization. This may be because an increase in AGDP raises domestic demand, reduces foreign exports and thus decreases the use of CAFTA. The distance variable is also negatively related to the CAFTA utilization, implying that the greater the distance and the higher the transport costs for foreign exports, the less incentive of the CAFTA utilization by ASEAN countries.

**Table 5:** Estimation results: ROO effect and margin effect.

Variables	(1)	(2)
	UR	UR
RI	-1.586***	-1.568***
	(0.0892)	(0.0754)
Margin	0	
	(.)	
MFN		1.098***
		(0.0326)
CGDP	6.503***	4.619***
	(0.2468)	(0.2096)
AGDP	-0.730***	-0.480***
	(0.0411)	(0.0340)
Distance	-2.006***	-1.182***
	(0.1093)	(0.0992)
Constant	-118.2***	-85.28***
	(5.6234)	(4.7937)
γ_ind_ * year_t_	-0.0171***	-0.0218***
	(0.0014)	(0.0012)
Observations	17367	40474
Adj. R-sq	/	/

The parentheses are standard errors. ***, **, and * show 1%, 5%, and 10% significance, respectively.

### 4.2. Robustness test

To ensure the robustness of the baseline regression results, we used a test that replaced the estimated model and selected the Probit model to estimate the equations. Table 6 shows the results of robustness test. The first column still shows that the tariff margin variable is omitted and the second column replaces tariff margin with MFN. We found that the RI variable is significantly negative at the 1% level and the MFN variable is significantly positive at the 1% level, indicating that rules of origin have a negative effect on the use of CAFTA, while tariff preference margin has a positive effect on the CAFTA utilization, which is consistent with the findings of the benchmark regression results. The signs and significance level of the remaining control variables are also in line with the benchmark regression. All of the above indicate that the baseline regression results are robust.

**Table 6:** Results of robustness test.

Variables	(1)	(2)
	UR	UR
RI	-0.907***	-0.849***
	(0.0498)	(0.0413)
Margin	0	
	(.)	
MFN		0.468***
		(0.0145)
CGDP	3.167***	1.906***
	(0.1196)	(0.0936)
AGDP	-0.327***	-0.197***
	(0.0188)	(0.0155)
Distance	-1.120***	-0.497***
	(0.0583)	(0.0489)
Constant	-56.68***	-34.67***
	(2.7197)	(2.1320)
γ_ind_ * year_t_	-0.00994***	-0.0104***
	(0.0008)	(0.0006)
Observations	17367	40474
Adj. R-sq	/	/

The parentheses are standard errors. ***, **, and * show 1%, 5%, and 10% significance, respectively.

### 4.3. Sample marginal effect analysis

In Table 5, the estimated results of column (2) show that the ROO effect is larger than the margin effect (in absolute terms). This means that the impact of ROOs on the use of CAFTA is much greater than that of tariff margin. To assess the specific effect of ROOs and tariff margin, we obtained the average marginal effects for all samples based on the regression result of Logit model. The average marginal effect coefficient of RI is -0.088, indicating that the restrictiveness index of ROOs increase one percentage point, the utilization rate of CAFTA will decrease by 8.8 percentage points. In contrast, the average marginal effect coefficient of tariff margin is 0.062, meaning that the tariff preference increases one percentage point, the utilization rate of CAFTA will increase 6.2 percentage points. Thus, it seems that rules of origin play an important role in promoting the use of CAFTA.

Based on the sample average marginal effect results, this study further calculates the relative contribution of two effects to the CAFTA utilization by each ASEAN country. We multiply the respective average marginal effect coefficients by the mean of the corresponding variables. For example, the contribution of the margin effect is calculated by the average marginal effect coefficient of tariff margin multiplied by the mean of corresponding samples. The contribution of each effect is calculated by exporting country and the results are reported in Table 7.

**Table 7:** The respective contribution to CAFTA utilization by exporting countries.

Country	Tariff margin	ROO Index
Brunei	0.1004	-0.2585
Cambodia	0.0053	-0.2656
Indonesia	0.1274	-0.2607
Laos	0.1291	-0.3030
Malaysia	0.1206	-0.2533
Myanmar	0.0049	-0.2954
Philippines	0.1219	-0.2527
Singapore	0.0647	-0.2378
Thailand	0.0538	-0.2602
Vietnam	0.0767	-0.2624

As can be seen from Table 7, the contribution of ROO effect to the CAFTA utilization by each country is larger than that of tariff margin effect to the CAFTA utilization. In particular, both tariff margin and ROO contribute the most to the use of CAFTA by Laos. What’s more, it is clear that ROO effect is greater than the tariff margin effect in column (2) of Table 5, similarly, the contribution of ROO effect is also more than that of tariff margin effect when calculating the relative contribution to the CAFTA utilization using the sample average marginal effect derived from the logit-based model. This indicates that the role of ROO is more significant than that of tariff margin in determining the use of CAFTA by each exporting country. This may be because the paper regards the products represented by HS 6-digit codes as the research subject, and rules of origin corresponding to different products vary, while tariff margins remain largely unchanged due to the achievement of zero tariffs for most products. Therefore, the impact of product-based rules of origin on the CAFTA utilization is more significant.

### 4.4. Heterogeneity analysis

In order to explore whether there is heterogeneity in the effects of ROO and tariff margin on the use of FTA, this paper divided the sample into three groups for heterogeneity analysis depending on the income level of the country. The h1, h2 and h3 represent lower middle-income, upper middle-income and high-income countries respectively. Moreover, the MFN is still used for estimation instead of tariff margin, and the estimated results are shown in Table 8.

**Table 8:** The estimated results of the heterogeneity analysis.

Variables	(1)	(2)	(3)
	UR(h1)	UR(h2)	UR(h3)
RI	-1.878***	-1.427***	-0.951***
	(0.1597)	(0.1030)	(0.3288)
MFN	1.128***	2.204***	2.196***
	(0.0756)	(0.0638)	(0.2053)
CGDP	4.443***	3.232***	4.151***
	(0.3162)	(0.2913)	(1.0269)
AGDP	-1.591***	1.387***	22.09***
	(0.1552)	(0.0845)	(2.4178)
Distance	-5.762***	-11.08***	-497.8***
	(0.6298)	(0.3143)	(55.9442)
Constant	-23.86***	-10.16	3656.6***
	(8.9613)	(7.2796)	(429.4716)
γ_ind_ * year_t_	-0.0265***	-0.0019	0.0157***
	(0.0028)	(0.0016)	(0.0046)
Observations	15839	19518	5117
Adj. R-sq	/	/	/

The parentheses are standard errors. ***, **, and * show 1%, 5%, and 10% significance, respectively.

The results show that the ROOs and tariff margin have a significant differential effect on the CAFTA utilization across countries with different income levels. As with the results in Table 5, ROO has a negative effect on the use of CAFTA while tariff margin has a positive effect on the CAFTA utilization. However, the ROO effect is larger than the margin effect only for lower middle-income countries, while the opposite is true for upper middle-income and high-income countries. This suggests that the ROOs have a greater impact on the use of CAFTA in lower middle-income countries, while the tariff margin has a greater impact on the use of CAFTA in upper middle-income and high-income countries. This is mainly due to the fact that upper middle-income and high-income countries have a higher share of exports to China than lower middle-income countries, and in turn obtain relatively higher benefits from tariff preferential treatment. Therefore, the effect of tariff margin on the CAFTA utilization when exporting from upper-middle and high-income countries is more significant.

### 4.5. Discussion

The study investigates the impact of ROOs and tariff margin on the use of CAFTA, Using the utilization rate as dependent variable to denote the use of CAFTA. The major explanatory variable is the restrictiveness index and tariff margin, which captures the effects of ROO and tariff margin on the CAFTA utilization, respectively. The results show that the effects of ROO and tariff margin is significant in affecting the use of CAFTA with expected sign.

First, the estimated coefficient of RI is negatively correlated with the CAFTA utilization, which indicates that the increasing of the restrictiveness index of ROOs can reduce the use of CAFTA. Based on a theoretical analysis of the cost effects of ROOs, the complex and stringent requirements under ROOs can increase the compliance cost of exporters from member countries, then offsetting the benefits from tariff preferential treatment. Consequently, the higher the degree of restriction of ROOs, the greater the costs imposed by ROOs, the lower the incentives for the CAFTA utilization, and then the exporters will reduce the use of CAFTA.

Second, we use the MFN tariff as a proxy of tariff margin, the estimated result shows that the tariff margin have a positive effect on the CAFTA utilization. We can infer from the results that the larger tariff margin can increase the use of CAFTA. The tariff margin represents the benefits that exporters derive from the CAFTA utilization. The larger the tariff margin, the greater the benefits obtained from the tariff preferential treatment, and thus the higher the likelihood of the CAFTA utilization by exporter.

Third, the study calculates the relative contribution of ROOs and tariff margin to the CAFTA utilization by exporting country using the sample average marginal effect. The results show that the ROO effect is larger than the tariff margin effect, indicating that the rules of origin play a more important role in the relative contribution to the CAFTA utilization by each ASEAN country. As mentioned above, since the establishment of the China-ASEAN FTA, zero tariffs have covered more than ninety percent of tariff lines on both sides, especially after the upgrade of the China-ASEAN FTA, which has accelerated the realization of zero tariffs on both sides’ products. The zero tariff on most products after the upgrade has resulted in smaller or even basically unchanged changes in tariff margin, so the tariff margin effect on the use of CAFTA is smaller. As the core provisions of the FTA, the ROOs assign different criteria to each product, and the stricter the criteria, the more restrictive it is, and thus the stronger the binding effect on the CAFTA utilization.

Four, based on the heterogeneity analysis, we found that ROO has a greater binding effect on the use of CAFTA in lower middle-income countries, and the tariff margin has a greater facilitating effect on the use of CAFTA in upper middle-income and high-income countries. This suggests that lower middle-income countries are more sensitive to the impact of ROOs, and a smaller increasing in the degree of ROO restriction could lead to a larger decrease on the use of CAFTA by lower middle-income countries. Unlike the lower middle-income countries, upper middle-income and high-income countries are more sensitive to the impact of tariff margin due to a higher share of exports, with a smaller increase in tariff margin could lead to a larger increase on the CAFTA utilization by upper middle-income and high-income countries. Therefore, ROOs play an important role in the use of CAFTA by lower middle-income countries, while the tariff margin plays an important role in the use of CAFTA by upper middle-income and high-income countries.

## 5. Conclusions

This study attempts to investigate the effects of ROO and tariff margin on the CAFTA utilization using the unbalanced panel data comprising of 40,474 product-year observations for China imports from the ASEAN countries from 2015 to 2021 under China-ASEAN Free Trade Agreement. The results show that rules of origin do have a negative effect on the use of CAFTA, while the tariff margin is just on the opposite. Also, the effect of the former is greater than the effect of the latter. Based on the sample marginal effects, we calculate the relative contribution of ROO effect and tariff margin effect to the CAFTA utilization by each ASEAN country. The results find that the relative contribution of ROO is higher than that of the tariff margin. That is, ROO plays an important role in determining the use of CAFTA by ASEAN exporters. Furthermore, the heterogeneity analysis reveals that ROO plays a greater role in the use of CAFTA by lower middle-income countries, and tariff margin plays a greater role in the use of CAFTA by upper middle-income and high middle-income countries.

As FTAs continue to proliferate, the utilization rate has become an important indicator to measure whether the implementation of FTA is effective. The lower utilization rate weakens the impact of FTA itself and reduces the economic effect of FTAs. As the core provisions of FTAs, rules of origin are originally designed to prevent free-riding by trade deflection, but overly stringent rules of origin reduce the benefits from tariff preferences by increasing the cost compliance for exporters, making them become a potentially hidden instrument for trade protection elaborated by Krishna and Krueger [32], this not only decreases the utilization rate of FTA, but also impedes the development of trade between countries. In particular, when the cost of meeting the rules of origin reaches a certain level, exporters will give up using FTA. In this way, the FTAs would have lost the meaning for which it was originally established. Therefore, this paper proposed some policy recommendations on how exporters can increase the use of FTA based on the findings above.

First, for the greater negative effect of ROO on the CAFTA utilization, we should reduce the degree of restriction by relaxing the criteria for determining the rules of origin. On one hand, designing and using the less restrictive single criteria or selective criteria rules, such as the criteria of change in subheading are easier for exporters to meet than the criteria of change in chapter. The selective criteria can reduce the degree of restrictiveness of rules of origin by increasing the flexibility of the exporter’s choice, thereby reducing the costs borne by the exporter to meet the rules of origin. On another hand, introducing more various regime-wide ROO provisions, which serve as an assistant instrument to the product-specific rules, it alleviates to some extent the degree of restrictiveness of ROOs, the most commonly used regime-wide provisions are the rules of de minimis and cumulation. Second, for the positive effect of tariff margin on the CAFTA utilization, we should accelerate the product coverage area of zero tariffs and promote 100 percent coverage of zero tariff products through further negotiations and consultations. Third, based on the differences in the effect of ROO and tariff margin on countries at different income levels, we should design more lenient rules of origin for lower middle-income countries, and should accelerate negotiations and implementation on tariff reductions for upper middle-income and high middle-income countries. Only in this way can ROO and tariff margin play their proper role in the FTA. The efficiency of the China-ASEAN FTA can be further enhanced by increasing the use of CAFTA, thus promoting sustainable development of bilateral trade.

The limitation of the study is that rules of origin include multiple determination criteria and the cost effects of different determination criteria vary. However, the impact of ROO on the FTA utilization is only analyzed as a dummy variable in this paper. Future research could group rules of origin with different criteria according to their degree of restriction, then analyze and compare their impact by introducing multiple dummy variables.

## Supporting information

S1 Data (XLSX)
